# Letter to Editor about “Peripheral nerve blocks for hip fracture surgeries: a bibliometric and visual analysis”

**DOI:** 10.1097/JS9.0000000000003125

**Published:** 2025-07-30

**Authors:** Mingxing Zhang, Zujian Xu

**Affiliations:** Department of Orthopedics, The Affiliated Traditional Chinese Medicine Hospital of Southwest Medical University, Luzhou City, China

*Dear Editor,*

We read with great interest your recently published article titled *“Peripheral nerve blocks for hip fracture surgeries: a bibliometric and visual analysis”*^[1]^. This study systematically reviewed English-language original research on peripheral nerve blocks (PNB) for hip fracture surgeries from 2004 to 2024. Utilizing tools such as VOSviewer and CiteSpace, the authors provided visual analyses of research trends, international collaboration networks, and keyword clustering. The article offers valuable insights for perioperative pain management researchers. However, we would like to raise a few considerations regarding the study’s design, analytical depth, and practical implications.

First, regarding the methodology, the study extracted data exclusively from the Web of Science Core Collection. While this database indeed includes a large number of high-quality English publications, the exclusion of non-English literature and other major databases (such as PubMed, Scopus, Embase, and CNKI) may have resulted in a narrower retrieval scope. Consequently, research from non-English-speaking countries with rapidly evolving PNB practices—such as Japan, South Korea, and France—may have been underrepresented, limiting the study’s global perspective.

Second, although the authors constructed several keyword clusters and identified research hotspots in techniques such as PENG and fascia iliaca blocks, the article could offer stronger clinical guidance. For example, it did not explore the differential applicability of various PNB techniques across surgical approaches (e.g., hemiarthroplasty vs. internal fixation) or specific patient populations (the very elderly or patients with dementia). Furthermore, while PNB was noted to facilitate early postoperative mobilization and reduce opioid use, the study did not compare these outcomes directly with other standard analgesic approaches (e.g., FICB, lumbar plexus block). This limits the ability to assess relative advantages or evidence strength from a bibliometric perspective.

Third, on the issue of technological equity and accessibility, the authors briefly noted that high-end ultrasound device costs may hinder broader PNB adoption but did not explore strategies for implementation in resource-limited settings. Prior studies have proposed alternatives such as nerve stimulator-guided techniques or simplified PNB approaches. Integrating bibliometric analysis with insights into technical barriers and dissemination strategies could enhance the paper’s value for anesthesia practice in lower-resource environments.

Lastly, we agree with the authors’ observation that systematic studies on mortality, long-term recovery, and cost-effectiveness related to PNB are still lacking. In summary, this article provides a comprehensive macro-level overview and quantitative mapping of PNB research in hip fracture surgeries. Nonetheless, it could be further strengthened by expanding data sources, enhancing clinical relevance, and offering more actionable recommendations for broader technical implementation.

We appreciate the authors’ contributions to this important field and look forward to future interdisciplinary and multicenter collaborations that promote the evidence-based and practical application of PNB.

## Data Availability

Not applicable.

## Research registration unique identifying number (UIN)

There are no conflicts of interest.

## Statement

The article was written in accordance with the TITAN Guidelines 2025^[2]^.
